# Pocket Mercury-Vapour Detection System Employing a Preconcentrator Based on Au-TiO_2_ Nanomaterials

**DOI:** 10.3390/s21248255

**Published:** 2021-12-10

**Authors:** Emiliano Zampetti, Paolo Papa, Andrea Bearzotti, Antonella Macagnano

**Affiliations:** Institute of Atmospheric Pollution Research–National Research Council (IIA-CNR), Research Area of Rome 1, Strada Provinciale 35d, 9, 00010 Montelibretti, Italy; p.papa@iia.cnr.it (P.P.); a.bearzotti@iia.cnr.it (A.B.); antonella.macagnano@cnr.it (A.M.)

**Keywords:** mercury, QCM, portable system, low cost, sensors, Arduino

## Abstract

In environments polluted by mercury vapors that are potentially harmful to human health, there is a need to perform rapid surveys in order to promptly identify the sources of emission. With this aim, in this work, a low cost, pocket-sized portable mercury measurement system, with a fast response signal is presented. It consists of a preconcentrator, able to adsorb and subsequently release the mercury vapour detected by a quartz crystal microbalance (QCM) sensor. The preconcentrator is based on an adsorbing layer of titania/gold nanoparticles (TiO_2_NP/AuNPs), deposited on a micro-heater that acts as mercury thermal desorption. For the detection of the released mercury vapour, gold electrodes QCM (20 MHz) have been used. The experimental results, performed in simulated polluted mercury-vapour environments, showed a detection capability with a prompt response. In particular, frequency shifts (−118 Hz ± 2 Hz and −30 Hz ± 2 Hz) were detected at concentrations of 65 µg/m^3^ Hg^0^ and 30 µg/m^3^ Hg^0^, with sampling times of 60 min and 30 min, respectively. A system limit of detection (LOD) of 5 µg/m^3^ was evaluated for the 30 min sampling time.

## 1. Introduction

Among the environmental gaseous pollutants harmful for human health, gaseous mercury is related, in several ways, to neurodegenerative human diseases [1]. Its effects have been extensively reported and described in many works [2,3]; therefore, it is important to monitor and understand the pathways of diffusion [4]. To adequately detect and quantify this element, both in outdoor or confined environments, there are different strategies and methodologies that can be used. The detection of gaseous mercury can be made with a real-time measurement, using reliable active instruments [5] or using passive samplers (PASs) [6,7], exposed for a given period and in a second quantified time. The measurements performed with active systems are, in most cases, hard to perform due to the instrument dimensions, and the difficulty in moving them to the site of interest. It should also be considered that these active systems require a source of electricity and gas cylinders for their operation. On the other hand, PASs are less accurate, due to short time measurements, but allow the possibility of being used in remote areas, giving a wider spatial resolution and providing a long-term trend for the pollution in the atmosphere [8]. This is possible due to their small dimensions, the facility of transportation and their simplicity of use.

In recent years, to overcome these difficulties in the sampling monitoring, research has been increasingly focused on the development of miniaturized portable sampling systems, that can conjugate the advantages presented by the PASs and the accuracy given by the active sampling instruments. An example is given by some portable analysers for detecting low or high mercury vapour concentrations, such as Lumex RA915M, Jerome 431-x, Gardis or Tekran 2537A instrumentations [9,10,11], which base their operating function on atomic absorption spectrometry with Zeeman background correction, gold film sensors, Cold Vapor Atomic Absorption Spectrometry (CVAAS) or Cold Vapour Atomic Fluorescence Spectrometry techniques. In some other case, the use of unmanned aerial vehicles (UAVs), have been developed to probe aloft, helping us understand the pollutant distribution in the atmosphere [12,13]. Following this trend, miniaturized portable systems will become increasingly crucial in the future for providing fast and easy information to personnel while operating in heavily mercury-polluted environments, with the aim of operating in environments that are safe for human health. Most miniaturized detection systems, used to detect very low concentrations with considerable sensitivity and accuracy in data, often rely on the use of a preconcentrator sampling system [14,15,16]. In fact, a preconcentrator is a fundamental attribute in low concentrations, increasing the minimum amount of pollutants necessary to be detected by the sensor. The preconcentration system also has a dual function; in addition to concentrating the analyte, it also eliminates or reduces the influence of possible interferents, discriminating the target gas in a selective way.

In the present study, a pocket-sized, fast and low-cost mercury-vapour detection system is presented. The system structure consists of two main parts. The first is based on a heater, which was covered by a sensing layer of titania/gold nanoparticles (TiO_2_NP/AuNPs) [7] suitable in the adsorption of mercury vapour acting as preconcentrator (PreHG), that desorbs once heated. The second consists of the sensor, in our case a 20 MHz gold-coated Quartz Crystal Microbalance (QCM), widely used in several application fields such as environmental, space, food, and biologic monitoring [17,18,19,20]. All air sampling pathways are connected to a pneumatic system which is, in turn, managed by a microcontroller. The reason behind the use of gold is due to its great affinity with mercury, which leads to the formation of an amalgam [21,22]. In our case, both the PreHG and the sensor were based on the gold–mercury amalgam mechanism [23]. All the steps, during the generation of the desired mercury-vapour concentrations and samplings, were submitted to the control of a reliable mercury-vapour analyser (Tekran^®^ model 2537A). This instrument uses a Cold Vapour Atomic Fluorescence Spectrometry (CVAFS) technique. It presents a limit of detection of 0.1 ng/m^3^, a high selectivity towards mercury vapour, a sensitivity <0.1 ng/m^3^ (for 5 min of sampling time) in the range of 1–1500 ng/m^3^, using the argon as a carrier gas with a consumption of 100 L/day, and overall dimensions of 58 cm × 48 cm × 23 cm (Tekran Corp., Toronto, ON, Canada). The performed tests involved the sampling and the quantification of defined mercury-vapour rates at different concentrations, and in a simulated mercury-polluted environment. Moreover, measurements in the presence of potential interfering elements, such as humidity, H_2_S or SO_2_ were performed. For these characteristics, this device provides short time responses, a low-cost construction and a very small pocket size. Its use can be employed especially in cases with relevant amounts of mercury-vapour concentrations that could be harmful for humans, the environment and wildlife.

## 2. Materials and Methods

### 2.1. System Description

**Figure 1:**
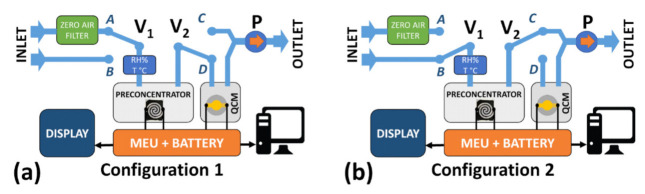
Schematic representation of the measurement system. Configurations C1 (**a**) and configuration C2 (**b**).

The whole measurement system is schematically represented in Figure 1a,b, where two operating configurations (C1 and C2) are shown. The core system consisted of two main parts. The first was based on a PreHG, where the adsorbent material was deposited; this is essential in the adsorption process of the mercury vapor. The second was the QCM sensor; this was necessary to detect and quantify the amount of mercury released by the PreHG after a certain sampling time. The flux of air sampled was regulated by a mini-DC membrane pump (model NMP03 by KNF), which was located downstream of the system. During the measurement the pneumatic pathway, the flux sample was managed by two electric valves (Series S070 by SMC), as seen in Figure 1 (V1, V2). As a result of these valves, it was possible to switch the flux in two pathways, and operate two configurations. In the first configuration (C1), Figure 1a, the valve V1 was connected to a filter (V1-A) where the environmental air was filtered, and passed firstly through the PreHG, then through the QCM sensor chamber (V2-D). In this configuration (Purge Mode), the flux air was regulated by the pump and set at 50 standard cubic centimeters per minute (sccm), in order to avoid possible influences of the flux air on the QCM surface. In this configuration the PreHG was heated, and the mercury released was sent to the QCM sensor chamber for detection and quantification. In the second configuration (C2), Figure 1b, the air flux was increased by the pump to 200 sccm, in order to sample an increased amount of air. The valves switched, sampling the air directly from the environment (V1-B position), while valve V2 sent the exhaust air to the pump (V2-C position). These two modes gave a clean air reference during the desorption/measurement (C1), and to avoided sending the stream of ambient air (to be analyzed) on the QCM during the sampling (C2).

**Figure 2:**
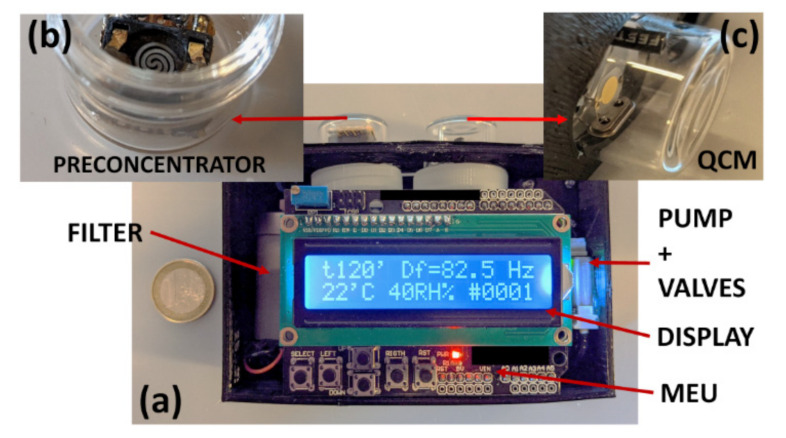
Pictures of the system prototype (**a**). A detail of PreHG (**b**) and QCM sensor (**c**) is reported. The overall dimensions are 90 × 111 × 45 mm with a weight of 250 gr.

During standard functioning, after power on, the system cleans the PreHg using C1 configuration. Successively, each measurement cycle is divided in two steps. The first uses C2 configuration to sample the air adsorbing the mercury vapour on the PreHG. The second uses C1 configuration to desorb the PreHG and to measure the released mercury by the QCM. The whole system was managed by a Main Electronic Unit (MEU), consisting of a microcontroller which regulated the sampling time, the desorption time, the acquired and saved data, the pump and the valve control. In Figure 2, a picture of the system prototype is reported with a detail of PreHG (Figure 2b) and the QCM sensor (Figure 2c).

The power supply was given by two Li-ion batteries (model 18,650, 3.7 V, 7800 mA), capable of powering the whole system continuously for 8 h.

### 2.2. Adsorbent Material

**Figure 3:**
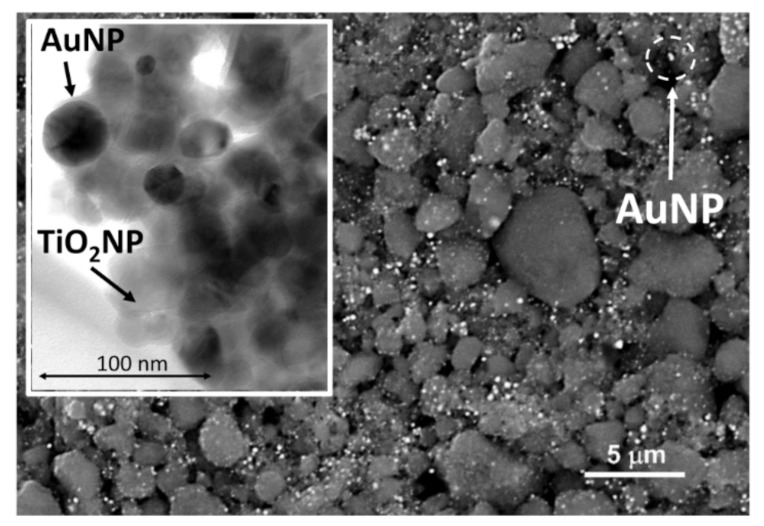
SEM-BSE micrograph of TiO_2_NP/AuNPs layer at 3500× magnification. The HR-TEM image is reported in the inset.

To collect and accumulate a considerable amount of mercury vapour on the PreHG, we directed our attention to a nanostructured adsorbent material [7,24], already widely tested and studied by our team in previous works, with applications in mercury vapour passive air samplers (PASs) [25]. The adaptability of this adsorbent material is given by its possibility to be used both in passive samplers (for slow samplings and long period of expositions) and in active sampling systems for short term samplings [26]. The adsorbent material consisted of a layer of titania nanoparticles, decorated with gold nanoparticles (TiO_2_NP/AuNPs). This material was synthesized, starting from the titania nanoparticles (anatase phase) suspended in a solution containing HAuCl_4_. Due to the photocatalytic properties of the titania, when subjected to a UV-light radiation, it led to the photoreduction of AuHCl_4_, forming gold nanoparticles on its surface. A polymer, polyvinylpyrrolidone (PVP), was used as capping agent and was removed by centrifugation. An aliquot of 3 µL of the TiO_2_NP/AuNPs water dispersion was poured onto the bare Pre-HG. The deposited material was characterized by scanning electron microscopy imaging (SEM) and with high resolution transmission electron microscopy (HR-TEM). In Figure 3, we report a backscattered electrons SEM image of the TiO_2_NP/AuNPs layer. Figure 3 confirms that the deposited material surface was arranged according to very rough nano and micro aggregates of the composite material.

In the SEM-BSE, the different brightness of the components, resulting from the backscattered electrons, highlights the aggregation of AuNPs (white or the brightest ones) in tiny spots, heterogeneously distributed onto the granular surface of TiO_2_ (dark grey). On the other hand, in the inset of Figure 3, the HR-TEM highlights the AuNP and the TiO_2_NP aggregation. Due to the high affinity of gold to mercury [21,27], this captures the mercury vapour that interacts with its surface with high efficiency, leading to the formation of an amalgam. Moreover, their nanometric dimensions have a high surface to volume ratio in the interaction with the analytes, showing a high absorption efficiency. During the tests, the influence of the temperature and humidity variability only slightly influenced the absorption mechanisms. For these reasons, this material fitted with the aim of the proposed system.

### 2.3. Preconcentrator

**Figure 4:**
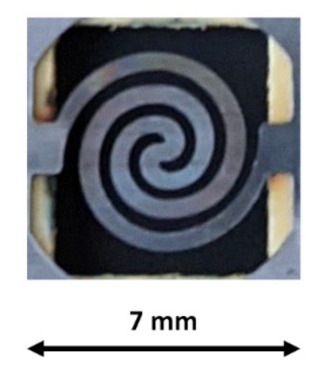
Representation of the bare Pre-HG, before the deposition of the sensing layer.

The PreHG consisted of a micro heater covered by the TiO_2_NP/AuNPs layer. The PreHG was a single spiral heating pattern, made of Ni-Cr, with a thickness of 150 µm [28], as shown in Figure 4.

**Figure 5:**
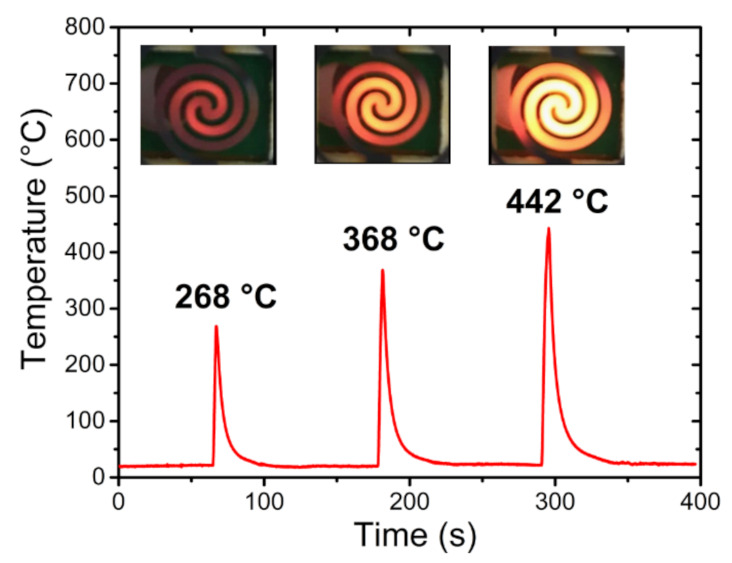
Heating intensity of the PreHG, both visually and by °C intensity, for the three heating times (2, 3 and 5 s).

The PreHG was previously tested without the adsorbent material, powered with 10 watts of direct current mode (DC) for different time lapses. Figure 5 reports the heater temperatures (T_heater_) directly measured by a thermocouple, kept in constant surrounding temperature (T_amb_) of 22 ± 2 °C, at three different heating times (2 s, 3 s and 5 s). To optimize the measurements, a high temperature silicon paste was used at a contact point between the head of thermocouple and the heating surface. As highlighted in Figure 5, the heater showed a fast increasing temperature with a longer powering time (t_pow_). In the same way, when the heater was turned off, the cooling time to reach the T_amb_ was in the order of tens of seconds. As shown in the lower part of the graph in Figure 5, the relative thermal visualization, for each heating time (2, 3 and 5 s), has been reported.

**Figure 6:**
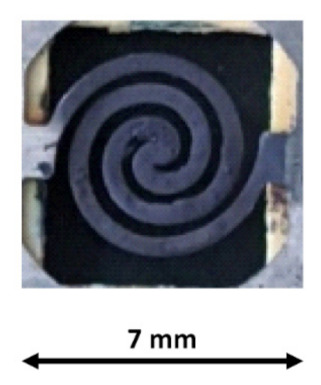
A picture of the PreHG after the deposition of the adsorbent material on its surface.

To deposit the adsorbent material (TiO_2_NP/AuNPs) on the PreHG surface, as seen in Figure 4, the drop casting technique was used, starting from the solution material previously prepared. Once 2 µL of solution was deposited with a micropipette, it was left to dry slowly in ambient air. Successively, a series of fast heating (lasting 5 s) at 440 °C was carried out to stabilize the deposited material. Figure 6 reports a picture of the PreHG covered by the adsorbent material.

In order to evaluate the suitable preconcentrator working temperature (to be used for all the successive measurements), a series of tests were performed. For this scope, the preconcentrator was exposed to different mercury vapour concentrations (up to 500 µg/m^3^), and then desorbed. All three heating times were tested. We exposed the preconcentrator, always at the same concentration and sampling time, to evaluate the subsequent amount of mercury released. A Tekran 2537A instrument was used for this. From these tests, we could evaluate a total mercury desorption after a double heating, with a heating time of 3 s (368 °C).

### 2.4. QCM Sensor

The sensor of the system consisted of a commercially available QCM, with a fundamental resonant frequency of 20 MHz and Au electrodes. The mercury, previously adsorbed by the preconcentrator, once released through a heating process, interacted with the QCM’s gold surface, leading to the formation of a second amalgamation process. The operation of a QCM sensor was based on the resonant frequency (*f_o_*), which was associated with a defined mass [29]. The mercury, adsorbed by the PreHG, once released through a heating process, interacted with the QCM’s gold surface, leading to the formation of an amalgamation process, changing the QCM mass. The mass variation induced a resonant frequency shift that can be calculated through Sauerbrey’s equation [30].

(1) Δf=−Cf ⋅f02⋅ΔmA

In this Equation (1), *f* refers to the frequency shift due to the changing mass m, *C_f_* represents the mass sensitivity constant, *f_0_* is the fundamental resonant frequency and *A* is the area of the interacting electrode. In order to evaluate the QCMs response variability, a batch of ten QCMs was tested, exposing them at the same mercury concentrations. Their changing frequencies were measured, connecting each QCM to a suitable oscillator circuit and a frequency counter (Racaldana with a resolution of 0.1 Hz). The measurement results showed a standard deviation of ±4 Hz.

### 2.5. Main Electronic Unit

Main electronic unit (MEU) consisted of a microcontroller, an oscillator circuit and a power regulator board. The microcontroller (µC) was a low cost 16-bit work of architecture (by Arduino) that managed all the measurements phases, controlled the heater activation, the valves and the pump, acquired the signal and display, and stored the data. A pulse-width modulation method was used to regulate the pump flux, reading the flux with a flow sensor (by Honeywell) that worked as a feedback element, while a PWM open-loop method was used to activate the heater. An important task of the µC was the QCM sensor frequency shift acquisition, due to mercury adsorption. An optimized oscillator circuit converted the QCM mass changes in a frequency shift that was acquired by a digital port of the µC. Environmental information, such as temperature (°C) and relative humidity (RH), were measured by an DHT22, connected to a µC serial bus. Finally, a power regulator board managed the battery charge and supplied the whole system.

### 2.6. Measurement Setup

**Figure 7:**
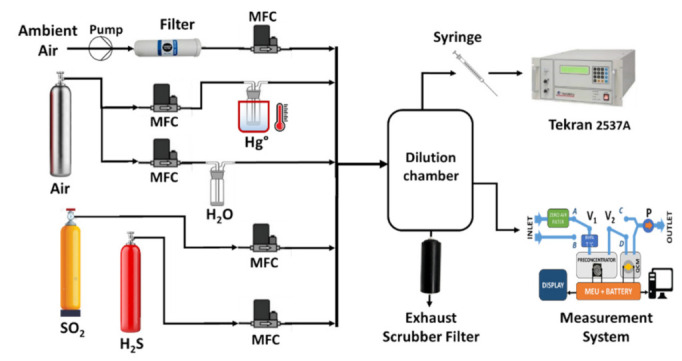
Depiction of the measurement setup and the instrumental system.

Figure 7 shows the configuration of the measuring system instrument. All the fluxes used were regulated through mass-flow controllers (MFC) from the MKS instrument, operating in sccm. The carrier gas was obtained using ambient air purified through a carbon filter, and two bubblers were used for the humidity and the mercury-vapour generator. Specifically, the mercury-vapour generator was kept in a thermal-regulated bath, to maintain the desired concentration constant. Finally, two certified gas cylinders (Rivoira S.r.l. certified, Milano, Italy), of 1.12 ppmv for the SO_2_ and 90 ppmv for the H_2_S were used for interfering gas tests.

All the desired mixed fluxes, generated by the MFC, were sent in a dilution chamber, with a capability of 5 L. This chamber was necessary to obtain homogeneity in the mixing of the gases. Subsequently, to verify the mercury-vapour generated concentrations, different withdrawn samples, operated by means of a gas tight syringe, were injected in the Tekran^®^ analyser 2537A. In this way it was possible to calculate and variate the desired concentrations of the dilution chamber. The Tekran^®^ analyser 2537A was previously calibrated by a primary calibration unit Tekran^®^ 2505. This ultra-precise and accurate closed vessel source of saturated gaseous mercury gas allowed calibration of the Tekran^®^ 2537A by injecting a defined amount of mercury vapour. The incoming flow in the dilution chamber was always greater than the flows sampled by the system, letting the excess flow out through an exhaust to a scrubber filter.

## 3. Results and Discussions

### 3.1. Mercury-Vapour Detection

**Figure 8:**
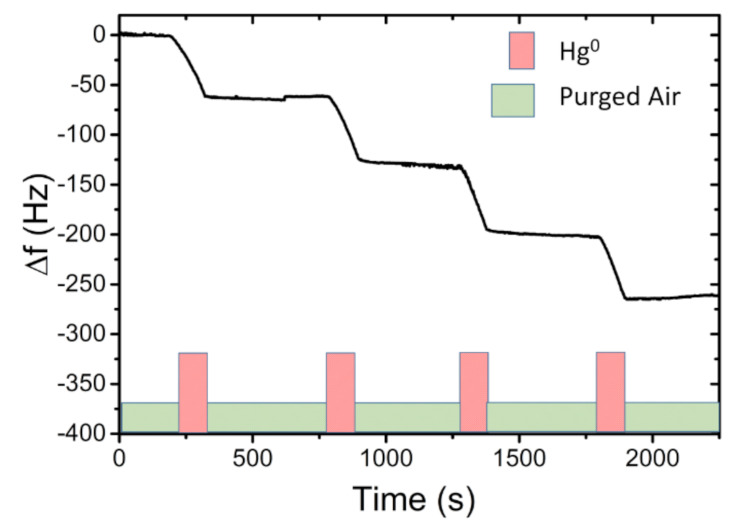
Cycles on-off of exposition at 6000 µg/m^3^ Hg^0^, lasting 2 min and then purged air.

Firstly, we valued the response of the QCM sensor when exposed to a flux of a defined mercury-vapour concentration. As previously shown in the setup scheme of Figure 7, for the mercury-vapour generation we used a bubbler, kept in a constant thermal-regulated bath; its exit was diluted by a carrier gas coming from filtered ambient air. These two gaseous fluxes were mixed and sent to a dilution chamber, where a desired concentration of 6000 µg/m^3^ ± 200 µg/m^3^ was obtained. For this test, from the dilution chamber, the system withdrew (by means of a pump) a flux of 50 sccm for 2 min, sending it directly to the QCM sensor (bypassing the preconcentrator chamber), alternating with purged air. Following a series of four expositions, a QCM frequency response was observed, as reported in Figure 8.

**Figure 9:**
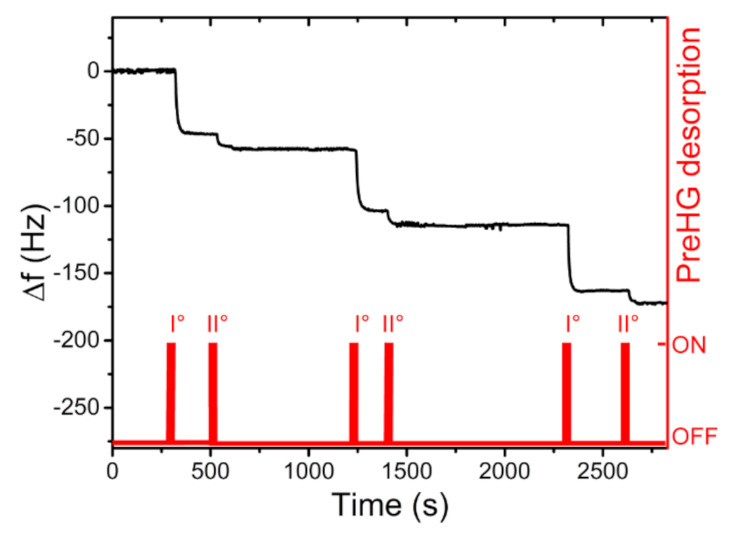
An example of mercury-vapour detection by QCM of three consecutive PreHG expositions and desorptions. The preconcentration (4 min each) happens after each double desorption, whereas the QCM remains in a stable state.

As shown by the graph in Figure 8, a total of four expositions (Hg^0^ EXP) are reported. Each EXP lasted 2 min, after that the flux was switched with purged air. During the QCM EXP, at a concentration of 6000 µg/m^3^ ± 200 µg/m^3^, a prompt response (0.5 Hz/s) was observed in the frequency shift (*f_out_ = f − f_0_*) with a total response of −60 Hz ± 2Hz for each EXP. During the switch with purged air, the QCM frequency remained approximately constant. This QCM behaviour confirmed the adsorption of the mercury on the Au electrodes, leading to the formation of an amalgam. After a series of measurements (with an overall accumulation of about −300 Hz), the QCM response capacity decreased linearly, so in order to restore the fundamental resonant frequency (*f_0_*) and avoid Au layer saturation, a thermal cleaning treatment in an oven at 150 °C for 30 min was performed. During this thermal cleaning treatment, the mercury amalgamated with gold was released and the QCM resonant frequency (20 MHz) was restored, without suffering damages. Successively, the preconcentrator adsorption capacity, towards polluted-air mercury, was tested. A vapour mercury concentration of 400 µg/m^3^ ± 10 µg/m^3^ was generated in the dilution chamber. The system withdrawn a flux of 200 sccm from the chamber, feeding it on the preconcentrator. Each sampling time, in C2 configuration, lasted 4 min, after that the system returned in C1 configuration. In C1, the preconcentrator was thermally desorbed, actuating the PreHG. The mercury vapour released by the PreHG was sent to the QCM sensor for the quantification. A series of three successive sampling and desorption measurements (exposition/desorption) were performed, as reported in the graph of Figure 9.

**Figure 10:**
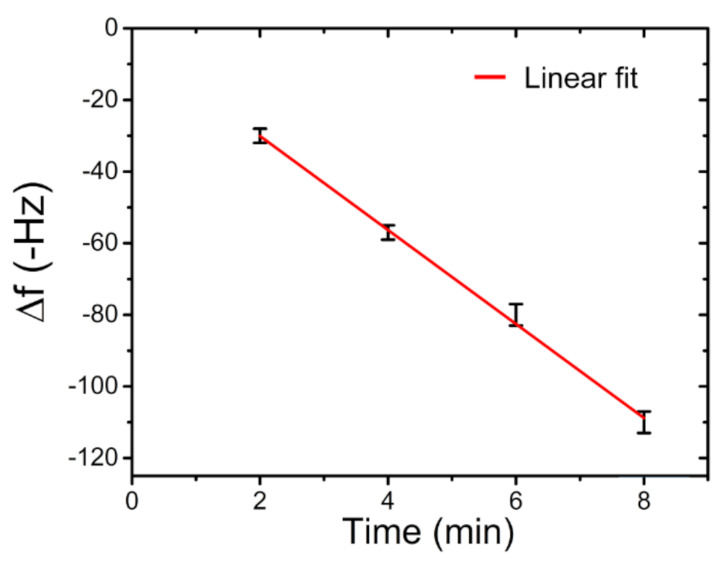
Frequency shift vs. exposition time at a mercury concentration of 400 µg/m^3^ ±10 µg/m^3^.

As highlighted on the graph in Figure 9, the mercury released from the preconcentrator was successively adsorbed by the QCM sensor, causing a negative frequency shift. Each desorption of the preconcentrator needed a double heating to obtain a total release of the adsorbed mercury. These measurements evidenced a good stability and repeatability response, with a mean frequency shift of −57 Hz and a maximum deviation of ±2 Hz, giving a response coefficient of about 13 Hz/min at this fixed concentration of 400 µg/m^3^ ± 10 µg/m^3^. Other measurements, with the same setup and the same mercury-vapour concentration, of 400 µg m^3^ ± 10 µg m^3^ were executed. Sampling times were set in 2, 4, 6 and 8 min. The frequency shift results of the four sampling times are reported in Figure 10.

**Figure 11:**
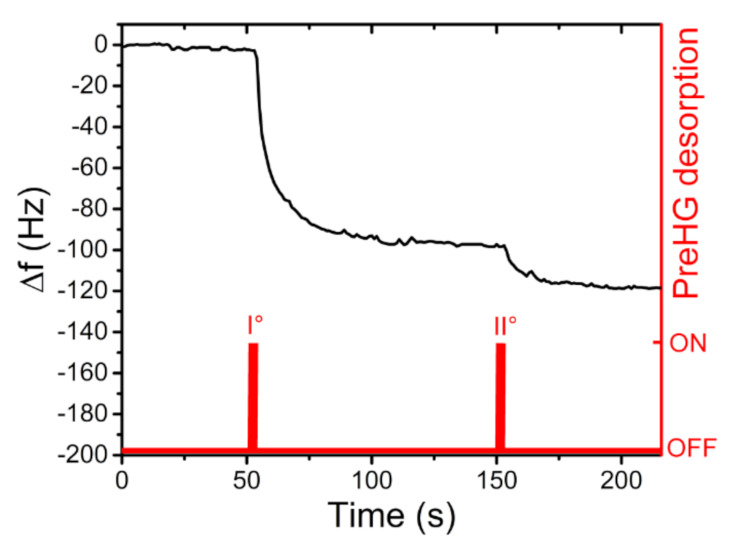
QCM frequency shift, following the desorption of the preconcentrator, after it was exposed for 60 min at a concentration of 65 µg/m^3^ of Hg^0^.

As highlighted in Figure 10, the system response shows a linear trend with an R2 of 0.997 and a sensitivity (S) of 0.034 Hz m^3^/µg min ± 0.003 m^3^/µg min, as defined in Equation (2):

(2) S=ΔfC⋅ts

where Δ*f* is the measured frequency shift (Hz), *C* is the mercury concentration (µg/m^3^) and *t_s_* is the sampling time exposition (min). The following measurements concerned the performances of the developed system when exposed in a context of a simulated mercury-polluted environment. Generally, in regard to human health, the permissible exposure limit (PEL) of the mercury-vapour concentration is 100 µg/m^3^ [31]. For these reasons, we set the mercury-vapour concentration in the dilution chamber at a value below this concentration. A successive concentration, of 65 µg/m^3^ ± 2 µg/m^3^ of Hg^0^, was generated, and performed with a sampling time of 60 min. During the desorption and subsequent mercury detection by the QCM, a total frequency shift of 118 Hz ± 2 Hz was detected, as seen in Figure 11.

**Figure 12:**
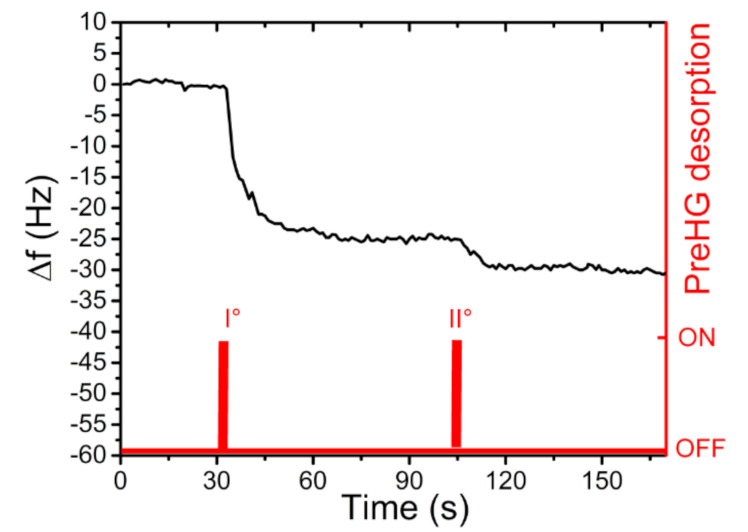
QCM frequency shift, after a preconcentrator exposition of 30 min, at a concentration of 30 µg/m^3^ of Hg^0^.

Furthermore, by decreasing the concentration in the dilution chamber to 30 µg/m^3^ ± 1 µg/m^3^ Hg^0^ and the exposure time to 30 min, a new measure was carried out. The relative results are reported in Figure 12.

Moreover, in this measure a distinct response was obtained, showing a frequency shift of 30 Hz ± 2 Hz, well above the background noise. In a real condition measurement, it could be possible to fix the time of the exposure (or air sampling time) at 30 min and after, using a calibration curve (frequency shifts vs. concentrations) to calculate the environmental concentration of mercury. The sampling period (30 min) can be used as the minimum time to have a valuable response in the range of 5 µg/m^3^ (LOD of proposed system) to 100 µg/m^3^ (human Permissible Exposure Limit).

### 3.2. Tests of Interferers

**Figure 13:**
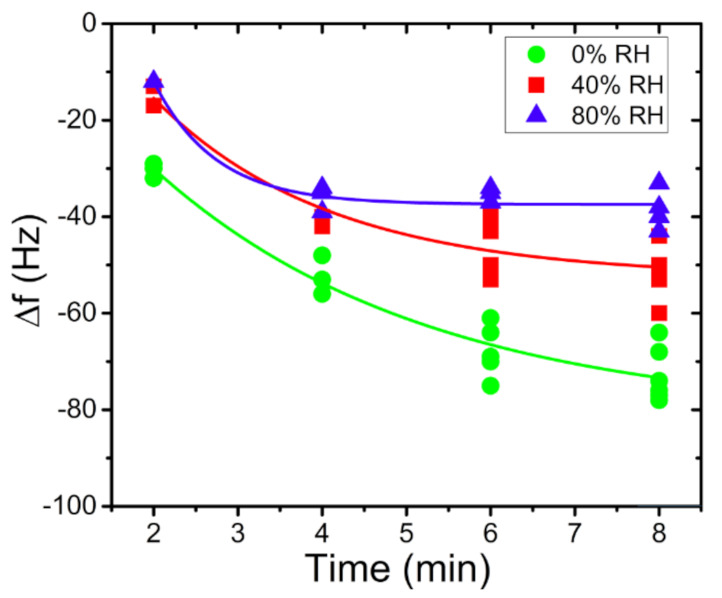
QCM frequency shift responses vs. sampling time at different humidity values and at fixed mercury concentration (400 µg/m^3^).

Following these measurements, further RH% tests in association with mercury-vapour expositions, testing preconcentrator adsorption capability in the presence of different humidity conditions, were performed. For these measurements, the preconcentrator was exposed for different lengths of time (2, 4, 6 and 8 min) and three different RH% concentration values (0, 60, 80 RH%), as seen in Figure 13. The sampling rate, as in the previous measurements, was constantly set at 200 sccm. In association with these parameters, a mercury-vapour concentration of 400 µg/m^3^ ± 10 µg/m^3^ was generated and kept constant for all measurements, checked with the Tekran^®^ analyser 2537A performing injections.

**Figure 14:**
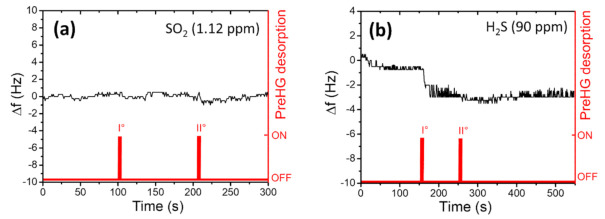
QCM frequency shift, after a PreHG exposition of 10 min, at 1.12 ppm of SO_2_ (**a**) and to 90 ppm of H_2_S (**b**).

As shown in Figure 13, the presence of high RH% values (blue points) interferes with the capacity of the PreHG in the mercury-vapour adsorption process. On the contrary, a higher mercury-vapour adsorption capacity by the preconcentrator was shown when dryer conditions were present. These tests helped to evaluate the effectiveness of the PreHG adsorption process, in the presence of humidity, in combination with mercury vapours. Clearly, the influence of the humidity can be related only to the adsorption process on the PreHG, and not on the QCM. In fact, in C1 configuration, both the PreHG and QCM chambers were fluxed with purged air, before proceeding with the desorption process. Beyond the humidity tests, other measurements regarding some interfering gases that could interact with the adsorption capacity of the PreHG during the sampling process were tested. Specifically, Sulphur Dioxide (SO_2_) and Hydrogen Sulphide (H_2_S) gases were tested. For these gases, we used two certified gas cylinders (Rivoira S.r.l. certified), of 1.12 ppmv for the SO_2_ and 90 ppmv for the H_2_S. A total flux of 200 sccm of interfering gas, was fluxed on the PreHG, for 10 min for each measurement. After the samples and the desorption processes, the QCM sensor did not evidence any significant response to the frequency shift for either interfering gases, as reported in Figure 14a,b. As highlighted in Figure 14a a non-valuable response was observed for SO_2_. Instead, a negligible response was observed for H_2_S, as reported in Figure 14b, taking into account a minimum signal to noise ratio, of three times the noise value (1.5 Hz).

Further considerations regarding the developed system showed a limit to the detection (LOD) value of 5 µg/m^3^, with an exposition time of 30 min. We determined the LOD with the method based on the noise value (in accordance with the International Council on Harmonization guidelines). According to our measurement results, we estimated the minimum detectable frequency value equal to 3 × noise (1.5 Hz) was about 5 Hz. From this value, and using the response of our instrument for a sampling time of 30 min (i.e., Figure 12), we found that the LOD was about 5 µg/m^3^. The autonomy of the system was calculated over 8 h of functioning, during a continuous mode sampling. Another important feature of this proposed system was the cost, related to the low-costs of the utilized components. In fact, the total estimated cost of the developed prototype was less than 1000 euros.

## 4. Conclusions

In the present work, we focused our efforts on the development of a portable pocket-sized instrument, capable of detecting and quantifying the mercury vapour. It has a low-cost production, an ease of use and a fast response in the presence of mercury-polluted environments that, according to the PEL (>100 µg/m^3^ Hg^0^), can be harmful for human health. This developed system consists of a preconcentrator based on nanomaterials, which adsorb and release mercury vapour after heat treatment. The detection of the released mercury vapour relied on gold electrodes 20 MHz QCM. The measurements showed a detectable frequency shift, of −118 Hz ± 2 Hz and −30 Hz ± 2 Hz, at concentrations of 65 µg/m^3^ Hg^0^ and 30 µg/m^3^ Hg^0^, with sampling times of 60 min and 30 min, respectively. The system showed an LOD of 5 µg/m^3^, evaluated over a 30 min sampling time. According to these measurements, a sensitivity of 0.034 Hz m^3^/µg min ± 0.003 m^3^/µg min was calculated. In the presence of relative humidity values of more than 40 %, the PreHG highlighted that Hg^0^ adsorption decreased, corresponding with a lower QCM response. Possible interfering gases, such as H_2_S and SO_2_ gave negligible signal results, at 90 ppm and 1.12 ppm, respectively. This pocket-sized mercury analyser has an autonomy of about 8 h in a continuous working mode. Finally, the total cost of this prototype is valued at less than 1000 euros. Additional enhancements can be developed in the system setup, and through the reduction of the influence of RH% for high values during the sampling.
